# Navigating Nutrition Information in the Digital Era: How Tourism and Recreation Students Evaluate Online Dietary Advice?

**DOI:** 10.3390/nu18193165

**Published:** 2026-09-25

**Authors:** Mateusz Rozmiarek

**Affiliations:** Department of Sports Tourism, Faculty of Physical Culture Sciences, Poznan University of Physical Education, 61-871 Poznan, Poland; rozmiarek@awf.poznan.pl

**Keywords:** nutrition, digital, social media, tourism, recreation, university, students

## Abstract

**Background/Objectives:** The rapid expansion of digital platforms has transformed the way individuals access, interpret, and apply nutrition-related information in everyday life. Although online sources provide convenient access to dietary knowledge, they also contribute to the spread of simplified, conflicting, and potentially misleading messages. Young adults, including university students, increasingly rely on digital platforms for health-related information; however, little is known about how students from non-medical fields evaluate the credibility of online dietary advice. This qualitative study aimed to explore how tourism and recreation students perceive, interpret, and assess online nutrition information in the digital era. **Materials and Methods:** A qualitative research design was applied using a semi-structured focus group discussion with 17 tourism and recreation students (9 women and 8 men), aged 21–23 years. The group discussion focused on students’ experiences with online nutrition information, criteria used to evaluate credibility, the perceived influence of social media influencers and online communities, and the role of academic background in shaping critical evaluation skills. The collected data were analyzed using thematic analysis to identify recurring patterns and key themes. **Results:** Four main themes were identified. Participants perceived online nutrition information as highly accessible and influential but also associated it with information overload, contradictory recommendations, and simplified explanations of complex nutritional concepts. Students evaluated dietary advice using multiple criteria, including source expertise, consistency across information sources, realistic expectations, explanatory quality, and social feedback. Social media influencers were primarily viewed as sources of inspiration and motivation, whereas professional sources were considered more reliable for important health-related decisions. Students also indicated that their academic background in tourism and recreation supported critical thinking and broader awareness of healthy lifestyles, although they recognized the limits of their nutrition-related expertise. **Conclusions:** Tourism and recreation students actively navigate a complex digital environment by combining information from professional sources, social media, online communities, and personal experiences. The findings highlight the importance of strengthening digital nutrition literacy and critical evaluation skills among young adults. Integrating elements of digital health literacy into university education may support more informed interpretation of online dietary advice and improve resilience against nutrition misinformation.

## 1. Introduction

Nutrition has become one of the most visible domains of health-related communication in contemporary society. Beyond its traditional role in disease prevention and health promotion, dietary behavior is increasingly associated with physical performance, body image, well-being, and lifestyle optimization [1,2,3]. This growing interest has been accompanied by a rapid expansion of digital sources of nutrition information, transforming how individuals acquire, interpret, and apply dietary knowledge in everyday life [4,5]. The internet, social media platforms, mobile applications, blogs, and online communities have created unprecedented opportunities for accessing nutrition-related content; however, they have also contributed to the dissemination of inaccurate, oversimplified, or commercially motivated dietary messages [6,7,8,9].

The digital transformation of health communication has fundamentally changed the relationship between experts, institutions, and information users. Previously, nutrition knowledge was predominantly transmitted through healthcare professionals, educational institutions, and traditional media [10,11]. Currently, individuals increasingly encounter dietary recommendations through decentralized digital environments where scientific evidence, personal experiences, marketing strategies, and popular trends coexist [12]. As a consequence, individuals are required not only to access information but also to evaluate its credibility, recognize potential misinformation, and make informed decisions regarding their own dietary practices.

This process is particularly important among young adults and university students, who represent a population characterized by intensive digital media use and increasing autonomy in lifestyle-related decision-making. University students commonly turn to online platforms when seeking information related to nutrition, weight management, physical activity, and health improvement [13,14,15]. At the same time, previous research indicates that young adults may experience difficulties distinguishing evidence-based nutritional recommendations from misleading online claims, particularly when information is presented in attractive, emotionally engaging, or personalized formats [16,17].

The increasing influence of social media has intensified challenges associated with nutrition communication. Platforms such as Instagram, TikTok, YouTube, and Facebook have become important spaces for sharing dietary advice, recipes, weight-loss strategies, and lifestyle recommendations [18,19,20]. Although these platforms may facilitate health education and peer support, they also enable the rapid spread of nutrition misinformation. Studies have shown that online dietary content often includes restrictive eating recommendations, unsupported claims regarding “detoxification,” unrealistic expectations regarding body composition, and simplified explanations of complex physiological processes [5,16,21,22]. Moreover, visual attractiveness and popularity indicators, such as the number of followers or likes, may influence users’ perceptions of credibility despite the absence of scientific validation [23,24,25].

Students enrolled in tourism and recreation programs represent an especially interesting group for investigating this phenomenon. Although these students are not necessarily trained as nutrition specialists, their academic and professional environment is strongly connected with health, physical activity, leisure behavior, wellness, and lifestyle promotion [26,27]. Professionals working in tourism, recreation, fitness, and wellness sectors increasingly interact with individuals seeking advice regarding healthy lifestyles, active leisure, and dietary practices. Consequently, future graduates may become intermediaries between health-related information and the broader public, making their ability to critically evaluate nutrition messages particularly relevant.

Understanding how this group evaluates nutrition-related information requires consideration of the broader evidence on how people interpret and assess dietary information in digital environments. Interview and focus-group research has shown that users assess online dietary information through factors such as source credibility, personal experience, perceived authenticity, and social interaction [17,28,29]. Qualitative studies have also highlighted the role of social media influencers and online communities in shaping how nutrition-related messages are interpreted, demonstrating that users may simultaneously value the accessibility and relatability of digital content while considering the credibility and reliability of the information encountered [28,29]. Other qualitative research has explored young adults’ experiences of nutrition information on social media, including how they interpret, evaluate, and incorporate potentially misleading or persuasive dietary content into their everyday lives [17,28]. However, much of the existing literature has focused on general young adult populations, social media users, or specific health and nutrition contexts, with relatively limited attention to how students from health- and lifestyle-related academic programmes themselves make sense of online dietary information. In particular, there is limited qualitative evidence examining how tourism and recreation students negotiate credibility, social influence, professional expertise, and their own academic background when evaluating online nutrition advice. The present study addresses this gap by using a focus group approach to examine not only individual perceptions but also how participants compare, question, and elaborate upon one another’s interpretations of digital nutrition information.

Although previous qualitative studies have provided valuable insights into experiences with online nutrition information, qualitative research remains less extensive than quantitative research in this area [30,31,32,33]. Quantitative surveys can identify levels of knowledge, attitudes, or behaviors; however, they provide limited understanding of the reasoning processes through which individuals determine whether online information is trustworthy. Focus group discussions allow researchers to explore personal interpretations, doubts, strategies of verification, and experiences with conflicting dietary messages. Such an approach is particularly appropriate for examining the complexity of contemporary digital health environments, where credibility is not determined exclusively by scientific accuracy but also by accessibility, familiarity, social validation, and perceived authenticity.

The aim of this qualitative study is to explore how tourism and recreation students evaluate and interpret online dietary advice in the digital era. The following research questions were formulated: (RQ1) How do tourism and recreation students perceive and describe online nutrition information available through digital platforms? (RQ2) What criteria do students use when evaluating the credibility and reliability of online dietary advice? (RQ3) What role do social media influencers, online communities, and professional sources play in shaping students’ dietary beliefs and behaviors? (RQ4) How does academic background in tourism and recreation influence students’ ability to critically evaluate online nutrition information?

The study was guided by several research assumptions derived from previous literature on digital health literacy, online nutrition communication, and health information evaluation. It was assumed that tourism and recreation students perceive online nutrition information as highly accessible while simultaneously recognizing challenges in distinguishing evidence-based recommendations from misinformation. Furthermore, it was assumed that students evaluate online dietary advice through multiple dimensions, including perceived scientific credibility, expertise of information providers, personal experiences, and social validation. Another assumption was that social media platforms influence students’ dietary perceptions not only through informational content but also through emotional appeal, visual presentation, and lifestyle identification. Finally, it was assumed that students engage in an ongoing negotiation process between professional nutritional knowledge, digital media narratives, and their own everyday experiences when making dietary decisions.

## 2. Materials and Methods

### 2.1. Research Design

This study employed a qualitative research design using a semi-structured focus group discussion (FGD) as the primary data collection method [34,35]. Qualitative approaches are particularly valuable when the objective is to explore perceptions, interpretations, and decision-making processes related to complex social and health-related phenomena that cannot be fully captured through quantitative measures [36,37]. Focus group discussions provide an opportunity to examine not only individual opinions but also the ways in which participants construct, negotiate, and reflect upon meanings through interaction with others.

Unlike individual interviews, focus groups allow researchers to observe how participants respond to shared topics, compare personal experiences, and develop collective interpretations of a phenomenon [34,35]. This approach was considered appropriate for the present study because online nutrition information is encountered within highly social digital environments, where opinions, recommendations, and beliefs are frequently shaped through interaction with peers, influencers, and online communities. 

### 2.2. Sample Selection

The study included 17 students enrolled in a Master’s degree programme in Tourism and Recreation at Poznan University of Physical Education, Poland. Participants comprised 9 women and 8 men, aged between 21 and 23 years. All participants were adults at the time of data collection and represented a relatively homogeneous educational group, sharing the same academic programme and level of study.

Participants were selected using purposive sampling. The decision to focus specifically on tourism and recreation students was intentional and reflected the exploratory aim of the study. This educational context was considered particularly relevant because tourism and recreation studies are connected with physical activity, leisure, wellbeing, health promotion, and lifestyle-related topics. Although the participants were not nutrition specialists, their academic background provided a relevant context for exploring how students with exposure to health- and lifestyle-related content perceive, interpret, and evaluate nutrition information encountered online.

The inclusion criteria were: (1) current enrollment in the tourism and recreation Master’s degree programme, (2) being 18 years of age or older, and (3) willingness to participate in a group discussion concerning online nutrition information and digital health communication. Participation in the study was entirely voluntary, and participants were informed that their participation or individual statements would have no academic or other consequences. The discussion was conducted outside compulsory class hours, was not connected with course assessment or grading, and participants could withdraw at any time without providing a reason.

The relatively homogeneous composition of the group was considered appropriate for the exploratory aims of the study and facilitated discussion within a shared educational context. At the same time, participants differed in their individual experiences, perceptions, and exposure to nutrition-related information, allowing both convergent and divergent perspectives to emerge. 

### 2.3. Focus Group Procedure

Although methodological guidelines frequently recommend focus groups consisting of approximately 6–12 participants to facilitate balanced interaction and in-depth discussion [38,39], the present study involved 17 participants. This decision was made because the study was designed as an exploratory examination of a relatively homogeneous academic cohort, and the researcher sought to capture a broad range of perspectives within a shared educational context. Conducting several smaller focus groups was considered less appropriate for the initial exploratory stage because the primary aim was to examine how participants collectively compared, challenged, and elaborated upon one another’s experiences within the same educational environment. A single group also allowed the researcher to observe interaction across a broader range of perspectives without introducing differences between groups that might have resulted from separate discussion dynamics.

The larger group size nevertheless had methodological consequences. A group of 17 participants necessarily provided less discussion time per individual than would have been available in smaller groups and may have limited opportunities for extended individual follow-up or more detailed exploration of some experiences. To address this issue, the discussion was actively moderated, with the researcher encouraging contributions from quieter participants, preventing individual participants from dominating the discussion, and using follow-up prompts when clarification or elaboration was needed. Participants were also encouraged to respond to and compare their experiences with those of other group members. This approach supported interaction and allowed both convergent and divergent perspectives to emerge.

The discussion lasted approximately one hour and was moderated by the researcher, who acted as a facilitator responsible for guiding the conversation, encouraging participation, maintaining balance between participants, and keeping the discussion aligned with the research questions. With participants’ consent, the discussion was audio-recorded and subsequently transcribed verbatim by the researcher. The transcript was checked against the recording to correct transcription errors and ensure accuracy. The relatively large group generated a broad range of perspectives across all four research areas, although the design may have reduced the depth of exploration of some individual experiences. This limitation was taken into account during the analysis and interpretation of the findings. 

A semi-structured discussion guide was developed based on the research questions and included four main thematic areas: (1) students’ experiences with online nutrition information and dietary advice; (2) criteria used to evaluate credibility and reliability of online sources; (3) perceptions regarding social media influencers, online communities, and professional nutrition sources; (4) the influence of academic background on critical evaluation of nutrition-related information. The discussion guide was not formally pilot-tested with a separate group of participants before the main focus group. Instead, the guide was reviewed by the researcher prior to data collection to ensure that the questions were clear, open-ended, non-leading, and directly aligned with the research questions and the four predefined thematic areas. The wording and sequence of the questions were designed to facilitate natural discussion while allowing the moderator to use follow-up prompts when participants introduced relevant issues requiring further clarification or elaboration.

The moderator used open-ended questions and follow-up prompts to encourage deeper reflection and clarification of participants’ statements. Participants were encouraged to share personal experiences, compare perspectives, and respond to opinions expressed by other members of the group. The researcher avoided directing participants toward particular answers and focused on facilitating an open discussion environment.

Given the academic context in which the discussion took place, the potential for social desirability bias was considered. Participants may have been inclined to present themselves as critical and informed consumers of online nutrition information. To minimize this possibility, the moderator emphasized the voluntary and non-evaluative nature of the discussion, used open-ended and neutral questions, and avoided indicating approval or disapproval of particular responses. Nevertheless, the possibility that the academic context influenced participants’ responses cannot be completely excluded and was considered during the interpretation of the findings.

The focus group discussion was conducted in Polish. For presentation in the manuscript, selected quotations were translated into English by the researcher. The translation aimed to preserve the semantic meaning, contextual nuances, and intended meaning of participants’ statements rather than to provide a strictly literal word-for-word translation. Where necessary, expressions were adapted to ensure readability in English while maintaining the original meaning.

All participants were informed about the purpose of the study, the voluntary nature of participation, and confidentiality procedures. Participants were asked to respect the privacy of other group members and not to disclose information shared during the discussion outside the group. The collected material was de-identified prior to analysis and reporting. As confidentiality among focus-group participants cannot be fully guaranteed, this limitation was explained to participants before the discussion.

### 2.4. Data Analysis

The collected material was analyzed using reflexive thematic analysis based on the framework proposed by Braun and Clarke [40]. This approach was selected because it provides a systematic yet flexible method for identifying, organizing, and interpreting recurring patterns within qualitative data.

The analysis followed several consecutive stages. First, the researcher became familiar with the discussion, identifying initial observations related to students’ perceptions of online nutrition information. Second, meaningful segments of the statements were coded inductively, focusing on issues related to accessibility of online information, credibility assessment, social influence, and educational background. Third, related codes were grouped into broader thematic categories, which were reviewed and refined to ensure internal coherence and clear distinction between themes. During this process, the researcher repeatedly checked whether emerging interpretations remained grounded in participants’ statements and whether potentially contradictory or divergent views had been adequately considered. Given the relatively large size of the focus group, the analysis also considered the interactive character of the discussion. Statements were interpreted not only as individual accounts but also in relation to participants’ responses to one another, including points of agreement, disagreement, clarification, and elaboration. This was particularly important for distinguishing perspectives that emerged independently from those that developed through group interaction. Finally, the identified themes were interpreted in relation to the research questions and the existing scientific literature.

The analysis was conducted by the researcher who moderated the focus group discussion. While this ensured familiarity with the contextual and interactive character of the discussion, the potential influence of the researcher’s position as moderator and analyst was explicitly considered during the analytical process. The researcher maintained reflexive notes throughout the analysis, documenting initial assumptions, interpretative decisions, changes to codes and themes, and issues requiring further consideration. These notes were used to support reflexive awareness and to distinguish participants’ statements from the researcher’s subsequent interpretations.

Methodological rigor was supported through an iterative process of comparing codes and themes with the original participant statements. Particular attention was paid to negative or divergent cases and to statements that did not fully fit the emerging thematic patterns. This process helped ensure that the final interpretation reflected the range of perspectives expressed during the discussion rather than only the most frequently occurring views.

## 3. Results

The thematic analysis of the focus group discussion identified four overarching themes that reflected how tourism and recreation students perceived, evaluated, and used online dietary information in their everyday lives. The following sections present each theme, illustrated with representative quotations from participants. Participant identifiers (P1–P17) are provided to indicate the distribution of perspectives across the focus group.

### 3.1. Perceptions of Online Nutrition Information

The discussion indicated that online nutrition information was highly accessible, constantly present, and closely integrated into students’ everyday digital routines. Participants frequently described the Internet as a primary source of quick information about dietary issues, mainly because of the convenience and speed of obtaining answers. As P2 explained, “Nowadays almost everything about diet can be found online. If I want to know something quickly, I usually search on Google or watch a short video on Instagram or TikTok”. Similarly, P10 highlighted that online sources were attractive because they provided simplified explanations that were easier to understand than academic materials: “Online information is easy to access and usually explained in a simple way, so it is more attractive than reading scientific articles”.

At the same time, the discussion revealed that accessibility was not perceived exclusively as an advantage. Participants also described the large volume of available information as overwhelming, particularly when different sources presented conflicting recommendations. P3 noted that “there is so much information that sometimes it’s hard to know what is true. One person says carbohydrates are bad, another says they are necessary”. This point was echoed by P15, who observed that although the Internet provides many perspectives, “this makes people confused because everyone seems to have different advice”. Thus, while participants valued the availability of multiple sources, they also recognized that the same abundance of information could increase uncertainty and make it more difficult to distinguish reliable recommendations from misleading ones.

The discussion further suggested that students did not perceive online nutrition information as uniformly positive or negative. Rather, their evaluations depended on how the information was presented and how easily its claims could be reconciled with other sources. Some participants appreciated the accessible and engaging format of digital communication, whereas others emphasized the risk that simplified messages might obscure the complexity of nutritional science. As P12 observed, “Social media makes nutrition look very simple, but I think in reality it is much more complicated than influencers present it”. This perception indicates that students were aware of a potential gap between the appealing format of online nutrition messages and the complexity of evidence-based dietary knowledge.

Another aspect raised during the discussion concerned passive exposure to nutrition content through social media algorithms. Rather than actively searching for dietary advice, participants frequently encountered nutrition-related posts, videos, and recommendations while using digital platforms for other purposes. P6 explained: “I think nutrition content online is everywhere. Even if I’m not looking for it, videos or posts about diets appear on my feed”. This observation reflects the increasingly embedded nature of nutrition communication within everyday digital environments, where dietary messages are encountered not only through intentional information seeking but also through routine social media use.

Importantly, the focus-group discussion indicated that participants were not simply passive recipients of this content. When conflicting information was discussed, participants referred to different ways of dealing with uncertainty, including comparing sources and considering the credibility of the person providing the information. Thus, the interaction between participants moved the discussion beyond the simple observation that online information was abundant and highlighted the strategies students used to navigate this environment.

### 3.2. Criteria Used by Students to Evaluate the Credibility of Online Dietary Advice

Participants described the evaluation of online dietary advice as a multidimensional process in which several factors influenced their perception of credibility. Rather than accepting digital nutrition content at face value, students reported using various strategies to assess whether information appeared reliable, including evaluating the expertise of the source, comparing information across different platforms, and identifying potentially unrealistic claims.

One of the most frequently mentioned criteria was the identity and professional background of the information provider. Participants indicated that they were more likely to trust dietary recommendations when they originated from qualified professionals or recognized institutions rather than anonymous users or social media personalities. As P8 explained, “First, I check who posted it. If it’s a dietitian or a university, I trust it more than a random influencer”. This suggests that perceived expertise and institutional affiliation functioned as important indicators of credibility when students encountered nutrition-related information online.

Another strategy reported during the discussion was cross-checking information using multiple sources. Participants described comparing different websites or posts before accepting a particular recommendation, especially when the advice concerned important dietary decisions. P7 stated, “I usually compare information from several funpages or reels. If different sources say the same thing, I feel more confident”. This approach indicates that students attempted to reduce uncertainty by looking for consistency between different sources rather than relying on a single online message.

The discussion also highlighted the importance participants attached to recognizing unrealistic or exaggerated promises. Students expressed skepticism toward dietary advice that suggested rapid transformations or guaranteed results. For example, P16 noted, “When someone promises very fast results, like losing ten kilos in two weeks, I don’t really believe it”. Similarly, P17 explained that “if advice sounds too perfect or too easy, I become suspicious because nutrition usually isn’t that simple”. These statements indicate that participants developed informal strategies for identifying potentially misleading content, particularly when recommendations contradicted their general understanding of healthy lifestyle principles.

Beyond evaluating who provided the information and what claims were made, participants also paid attention to the way dietary advice was explained. P4 described the importance of supporting explanations: “I pay attention to whether the person explains why something works, not only says that it works”. This reflects an emerging awareness that perceived credibility was related not only to the message itself but also to the presence of reasoning or evidence supporting the recommendation.

The role of social feedback generated a somewhat more nuanced discussion. Some participants referred to comments and user experiences as supplementary cues when assessing online dietary information, while recognizing that such feedback could not establish scientific accuracy. P1 explained, “Sometimes I look in the comments because people often write if something helped them or if it seems fake”. The discussion suggested that social validation could complement, but did not necessarily replace, other credibility criteria such as professional expertise and consistency with information from additional sources.

This distinction was important because participants appeared to differentiate between information that was perceived as personally convincing and information that was considered scientifically reliable. Thus, credibility was not treated as a single characteristic but rather as an assessment based on several overlapping cues.

### 3.3. The Role of Social Media Influencers, Online Communities, and Professional Sources in Shaping Dietary Beliefs and Behaviors

Participants described social media as an important environment influencing their perceptions of nutrition and everyday dietary practices. However, the discussion indicated that the role of digital sources was not uniform. Influencers and online communities were often perceived as accessible and engaging sources of inspiration, whereas professional sources were generally considered more reliable when making important health-related decisions. At the same time, participants recognized that the more engaging format of influencer content could make it more attractive than professional communication.

A recurring theme was that social media influencers could influence dietary behaviors indirectly by providing practical inspiration rather than direct recommendations to follow specific diets. Participants often described influencers as sources of meal ideas, recipes, or motivation to adopt healthier habits. P9 explained that “influencers give me ideas for meals or recipes, but I wouldn’t change my whole diet because of one video”. Similarly, P5 noted that “sometimes influencers motivate me to eat healthier or cook more instead of buying fast food”. These statements suggest that participants perceived influencer content as useful in shaping everyday behaviors, particularly when it involved simple and achievable lifestyle changes rather than strict dietary rules.

At the same time, participants recognized that the persuasive power of influencers was connected not only to the information they shared but also to how they presented themselves. Students pointed out that visual aspects, personal stories, and lifestyle demonstrations could increase the perceived attractiveness and trustworthiness of online figures. P11 observed that “many people trust influencers because they show their own body and lifestyle, so it looks convincing”. This suggests that students were aware that perceived credibility in digital environments could be influenced by personal image and perceived authenticity rather than solely by professional expertise.

The discussion of online communities revealed a similar tension between usefulness and reliability. Participants appreciated the opportunity to learn from other people’s experiences; however, they also recognized that personal recommendations might not be universally applicable. P13 explained, “online groups can be helpful because people share experiences, but everyone has a different body, so their advice doesn’t always work”. This indicates that participants distinguished between experiential knowledge shared within online communities and evidence-based nutritional guidance.

The contrast between accessibility and authority became particularly apparent when participants discussed professional sources. Participants generally associated health professionals with greater expertise and reliability than popular online personalities. P14 stated, “if I have an important health question, I would rather listen to a dietitian than someone popular on Instagram”. However, this preference did not mean that professional sources were always perceived as the most attractive or engaging. As P8 explained, “professional sources seem less interesting because they are more formal, while influencers explain things in a way that is easier to understand”.

Taken together, the discussion suggested that participants did not evaluate digital sources according to a simple hierarchy. Instead, they distinguished between different functions of online content: influencers could provide motivation, practical ideas, and relatable examples, whereas professionals were preferred when students perceived a need for authoritative information. This distinction emerged through the discussion of the relative strengths and limitations of different types of sources.

### 3.4. The Influence of Academic Background on Students’ Ability to Critically Evaluate Nutrition-Related Information

Participants indicated that their academic background in tourism and recreation influenced the way they approached online nutrition information, primarily by increasing awareness of health-related issues and encouraging more critical evaluation of digital content. Importantly, participants did not describe their education as providing specialized nutritional expertise. Rather, they viewed it as supporting broader critical thinking and health literacy.

Several participants emphasized that university education contributed to a more reflective approach toward online information. P3 stated, “Our studies make me think more critically”. Another participant, P15, explained that although nutrition was not a major component of the curriculum, their education provided sufficient knowledge to identify questionable recommendations: “We don’t study nutrition in detail, but I think we know enough to notice when advice sounds unrealistic”. These statements suggest that participants perceived their academic background as supporting general critical evaluation rather than providing professional nutritional competence.

Participants also described a change in their approach to online dietary advice over time, particularly after entering university. P14 reflected that “before university I believed almost everything I saw online. Now I usually think twice before following dietary advice”. This account suggests that higher education may contribute to a more cautious interpretation of health-related information. However, participants also acknowledged that greater awareness did not eliminate uncertainty, particularly when they encountered claims requiring specialized nutritional or medical knowledge.

Another aspect discussed by participants was the broader perspective on health developed through tourism and recreation studies. Students emphasized that their education encouraged them to view healthy lifestyles as a combination of multiple factors, including physical activity, nutrition, recovery, and everyday habits. P13 explained that “subjects related to physical activity and health helped me understand that there is no one perfect diet for everyone”. Similarly, P11 noted that “our education encourages us to look at health from a broader perspective, not only through diet but also physical activity, sleep and everyday habits”. These observations indicate that participants understood nutrition within a wider health-promotion framework rather than as an isolated aspect of wellbeing.

The discussion also revealed clear boundaries to the perceived influence of academic education. Participants distinguished between being more capable of questioning general dietary claims and possessing professional nutritional knowledge. P1 stated, “I still don’t feel like an expert, but I know it’s important to check information before sharing it with other people”. Similarly, P3 explained, “studying tourism and recreation makes me more aware of healthy lifestyles, but if I had a medical problem, I would still ask a specialist”. These statements demonstrate that participants perceived their academic background as increasing critical awareness while simultaneously recognizing the limits of their own expertise.

Overall, the focus-group interaction suggested that academic background was viewed less as a source of specialized nutrition knowledge and more as a factor supporting critical thinking, awareness of the complexity of health, and greater caution when evaluating online dietary information.

## 4. Discussion

The present exploratory qualitative study examined how a group of tourism and recreation students perceived and interpreted online dietary advice in the digital era. The findings suggest that, within this particular group, students viewed online nutrition information as an important component of everyday health-related decision-making while also recognizing challenges associated with information overload, conflicting recommendations, and the difficulty of distinguishing evidence-based knowledge from potentially misleading content. Rather than indicating how university students in general evaluate online dietary information, the findings provide a context-specific account of how these 17 participants described their experiences, perceptions, and strategies. The study therefore contributes to the growing literature on digital health communication by illustrating how participants in one academic context described their navigation between different sources of knowledge, including professional expertise, social media content, peer experiences, and personal observations.

The increasing importance of online nutrition information reflects broader transformations in health communication. Digital platforms have changed the traditional relationship between experts, institutions, and information users by allowing individuals to directly access, share, and evaluate health-related knowledge [10,12]. Contemporary users are no longer only recipients of health information but also participants in its circulation through social networks and online communities [41,42]. The present findings are consistent with this broader perspective, as participants described online nutrition information as highly accessible and integrated into everyday digital routines. Similar observations have been reported among young adults, who increasingly rely on online sources and social media platforms when searching for health-related information [18,42,43]. However, given the exploratory nature of the present study and its context-specific sample, these findings should be interpreted as illustrative rather than representative of university students more broadly.

However, accessibility does not necessarily guarantee reliability. Participants frequently described the online nutrition environment as overwhelming due to the large volume of available information and the presence of contradictory dietary recommendations. This finding corresponds with previous research describing the digital food environment as a complex space where scientifically supported information coexists with commercial messages, personal opinions, and misinformation [5]. Nutrition represents a particularly vulnerable area of online communication because dietary behaviors are closely connected with identity, body image, lifestyle aspirations, and personal beliefs [1,15]. As a result, nutrition-related messages are often presented in emotionally engaging and simplified formats that may increase their appeal while reducing scientific complexity.

The students’ descriptions also align with previous research on online health misinformation. Systematic reviews have demonstrated that misleading health-related information is widespread across social media platforms and may include unsupported claims, exaggerated outcomes, and recommendations lacking scientific evidence [6,44]. Recent studies focusing specifically on nutrition misinformation have highlighted the prevalence of simplified dietary narratives, restrictive approaches, and unsupported claims related to health improvement and body transformation [5,8,16]. Importantly, the present study suggests that students were aware of these challenges and attempted to critically evaluate information rather than accept online recommendations without reflection.

One of the most important findings was that participants used multiple criteria when assessing the credibility of online dietary advice. They considered the qualifications of information providers, consistency between different sources, explanation of mechanisms, realistic expectations, and social feedback. These findings correspond with previous conceptualizations of digital health literacy, which emphasize that effective use of online health information requires the ability not only to locate information but also to evaluate its quality and relevance [45,46]. Research among university students has similarly indicated that eHealth literacy includes skills related to searching, understanding, and critically assessing digital health information [43].

The reliance on source credibility observed in this study is also consistent with research demonstrating that users frequently apply credibility heuristics when evaluating online information. In digital environments, perceived expertise, institutional affiliation, reputation, and trustworthiness often influence whether information is accepted as reliable [47,48,49]. Previous studies have shown that users may rely on easily observable indicators, such as popularity, social endorsement, or engagement metrics, when assessing online health messages [23,50]. Although such strategies may help individuals navigate large volumes of information, they may also create risks because popularity does not necessarily reflect scientific validity.

The role of social media influencers represents another important contribution of this study. Participants did not perceive influencers exclusively as sources of misinformation; rather, they described them as providers of practical inspiration, motivation, and lifestyle ideas. This finding supports previous research suggesting that social media influencers increasingly participate in health communication by combining informational content with personal narratives, authenticity, and emotional engagement [51]. In particular, visual presentation and perceived similarity between influencers and audiences may increase trust and persuasive impact [52,53].

At the same time, participants demonstrated awareness that influencer-generated content should not automatically replace professional advice. They distinguished between influencers, who provide accessible and engaging lifestyle content, and professionals, who represent expertise and scientific authority. This distinction is consistent with studies showing that individuals often combine multiple sources of health information, using informal sources for inspiration while relying on professional sources for more important decisions [48,54]. Such findings suggest that the influence of social media on dietary behavior is not simply a question of whether users trust influencers, but rather how different sources are integrated within personal decision-making processes.

The academic background of tourism and recreation students provides an additional perspective on these findings. Participants perceived their education as contributing to greater awareness of healthy lifestyles, physical activity, and health promotion. Previous research among tourism and recreation students has emphasized the relationship between this educational field and lifestyle-related competencies, including physical activity promotion and health-oriented behaviors [26,27]. In the present study, participants similarly described their academic background as helpful when approaching online health and nutrition information. However, these accounts represent participants’ perceptions of their own educational experiences and do not demonstrate that the tourism and recreation programme objectively improves digital nutrition literacy. Consequently, the finding should be understood as an exploratory observation concerning how students themselves interpret the role of their academic background rather than as evidence of an educational effect.

This distinction is particularly important because the study did not include students from other academic disciplines and did not objectively assess participants’ ability to distinguish accurate from inaccurate nutrition information. Participants also recognized the boundaries of their expertise. They described greater awareness and a more cautious approach to online information but did not perceive themselves as nutrition specialists. This distinction is consistent with the broader concept of health literacy, which emphasizes the ability to access, understand, appraise, and use health information without requiring individuals to become specialists [46,55]. Nevertheless, whether the educational context of tourism and recreation actually contributes to these competencies remains an empirical question that requires comparative and longitudinal research.

## 5. Study Limitations and Directions for Future Research

The present study has several limitations that should be considered when interpreting the findings. One important limitation concerns the generalizability of the findings. The qualitative design limits the possibility of statistical generalization. The study included 17 tourism and recreation students, and therefore the findings should not be interpreted as representative of all university students or young adults. However, the aim of qualitative research is not to achieve population-level generalizability but rather to provide an in-depth understanding of individual experiences, perceptions, and meaning-making processes.

Another limitation relates to the use of a single focus group involving all 17 participants. Although the relatively large group size facilitated the exchange of diverse perspectives and enabled participants to compare experiences and respond to one another’s views, the use of only one focus group limits the depth and transferability of the qualitative findings. In particular, conducting a single group does not allow formal claims of data saturation to be made, as additional focus groups might have revealed further perspectives, divergent experiences, or alternative interpretations. The dataset was nevertheless considered sufficient for the exploratory aims of the present study because all four research areas were actively discussed and the analysis resulted in four coherent themes grounded in participants’ statements. Importantly, the findings should be understood as exploratory and context-specific rather than as evidence of thematic saturation across the broader population of tourism and recreation students. Future research should involve multiple focus groups to examine whether the identified themes are replicated, expanded, or challenged in other participant groups.

The focus-group format also introduces specific limitations related to group dynamics. Group interaction may have influenced the extent to which participants expressed particular views. Conformity effects may have occurred when participants adjusted or moderated their opinions in response to dominant or commonly expressed perspectives within the group. Similarly, social desirability may have influenced participants’ accounts, particularly because the discussion concerned health, nutrition, and the evaluation of online information, topics for which participants may have wished to present themselves as informed and critical consumers. Although the moderator encouraged balanced participation and invited participants to express different or opposing views, more vocal participants may nevertheless have had greater influence on the direction and content of the discussion. Conversely, some participants may have been less willing to disclose personal experiences or opinions in front of their peers, potentially limiting the depth of individual accounts. These dynamics are particularly relevant given that all participants took part in a single group and shared a common educational context. Consequently, some perspectives may have been reinforced through group interaction, whereas others may have remained underrepresented. Future research using multiple smaller focus groups, individual interviews, or a combination of qualitative methods could provide greater opportunities for individual disclosure and help determine whether the findings are consistent across different group dynamics.

The recruitment of participants from a single tourism and recreation programme at one university further limits the transferability of the findings. Tourism and recreation students may have particular exposure to topics related to physical activity, leisure, wellbeing, health promotion, and lifestyle practices, which could shape their awareness of nutrition-related issues and their approaches to evaluating online dietary information. At the same time, the curriculum, educational experiences, and institutional context of a single programme may have contributed to shared ways of interpreting health and nutrition information that would not necessarily be present among students from other academic disciplines or institutions. Consequently, the findings should be interpreted as context-specific rather than representative of tourism and recreation students more broadly. Future studies could include participants from multiple universities and academic backgrounds, including health sciences, social sciences, engineering, and humanities, as well as participants from different cultural or national contexts, to examine whether the identified patterns are consistent, modified, or challenged across different educational, institutional, and cultural settings and to better assess the role of educational background in digital nutrition literacy.

Another consideration concerns the reliance on self-reported experiences obtained through the focus group discussion. Participants described their perceptions, attitudes, and strategies regarding online dietary information; however, their reported behaviors may not always correspond to their actual online practices. Future research combining qualitative focus group discussions with observational methods, digital trace analysis, or experimental designs could provide additional insights into how individuals interact with nutrition-related content in real online environments.

Finally, the study focused primarily on students’ perceptions of online dietary advice rather than objectively assessing the accuracy of the information they encountered. Although participants demonstrated various strategies for evaluating credibility, future studies could examine the relationship between perceived credibility and the actual scientific quality of online nutrition content. Such approaches may contribute to a better understanding of whether individuals’ evaluation strategies effectively protect them from misinformation.

## 6. Practical Implications

The study provides several important practical implications. The findings indicate that university students operate within a complex digital nutrition environment where accessibility, social influence, professional expertise, and personal experience interact in shaping dietary beliefs and behaviors. Therefore, improving nutrition-related decision-making should not focus solely on providing more information but should also emphasize the development of critical evaluation skills and digital health literacy.

Although the present study cannot establish the effectiveness of any educational intervention, the participants’ accounts point to the potential value of incorporating digital nutrition literacy into university education. This may be particularly relevant in fields related to health promotion, physical activity, tourism, recreation, and lifestyle studies, although the relevance and effectiveness of such educational approaches should be evaluated empirically across different academic contexts. Digital nutrition literacy could be addressed through dedicated modules, workshops, or practical learning activities focused on identifying reliable sources, recognizing misleading or exaggerated claims, understanding scientific uncertainty, distinguishing evidence-based recommendations from personal opinions, and critically evaluating nutrition-related content on social media. Students could, for example, analyse real-world social media posts, compare influencer recommendations with evidence-based guidelines, assess the credibility of different online sources, and discuss how commercial interests and persuasive communication techniques may influence the presentation of dietary information. Such approaches represent potential educational strategies rather than interventions demonstrated to be effective by the present study.

The integration of digital nutrition literacy could also be supported through interdisciplinary collaboration between nutrition specialists, public health educators, communication specialists, and academic staff representing fields in which health and lifestyle topics are commonly addressed. Importantly, such education does not require students to become nutrition specialists but may aim to develop competencies necessary for responsible interpretation of health information and informed decision-making in digital environments.

The findings also have implications for nutrition professionals and public health institutions. Since participants perceived professional sources as reliable but sometimes less engaging than influencer-generated content, health communication strategies should consider adapting scientific messages to contemporary digital formats. Clear, accessible, and visually engaging communication may increase the reach and effectiveness of evidence-based nutrition information, particularly among younger audiences.

Finally, the study highlights the need for cooperation between nutrition experts, educators, and digital communication specialists. In an environment where dietary information is increasingly shaped by social media platforms and online communities, effective health promotion requires not only correcting misinformation but also creating trustworthy, understandable, and engaging alternatives. Supporting individuals’ ability to critically evaluate online nutrition content may therefore represent a promising area for future educational and public health research on dietary decision-making in the digital era.

## 7. Conclusions

This qualitative study provides insights into how tourism and recreation students evaluate and interpret online dietary advice in the digital era. The findings indicate that students perceive online nutrition information as an easily accessible and influential resource, but they also recognize challenges related to information overload, contradictory recommendations, and the presence of potentially misleading content. Participants described their evaluation of dietary advice as involving a combination of source credibility assessment, comparison of information, recognition of unrealistic claims, and consideration of social influences. Social media influencers and online communities were perceived as important sources of inspiration and motivation, whereas professional sources remained the preferred reference point for more important health-related decisions. Participants also perceived their academic education in tourism and recreation as helpful in developing a broader perspective on lifestyle, wellbeing, and health promotion, although they recognized that it does not replace specialized nutrition training. These findings highlight the need to strengthen digital nutrition literacy among young adults by supporting their ability to critically evaluate, interpret, and apply nutrition information within increasingly complex online environments.

## Data Availability

The original contributions presented in the study are included in the article; further inquiries can be directed to the corresponding author.
